# A small RNA mediated regulation of a stress-activated retrotransposon and the tissue specific transposition during the reproductive period in *Arabidopsis*

**DOI:** 10.3389/fpls.2015.00048

**Published:** 2015-02-09

**Authors:** Wataru Matsunaga, Naohiko Ohama, Noriaki Tanabe, Yukari Masuta, Seiji Masuda, Namiki Mitani, Kazuko Yamaguchi-Shinozaki, Jian F. Ma, Atsushi Kato, Hidetaka Ito

**Affiliations:** ^1^Faculty of Science, Hokkaido University, SapporoJapan; ^2^Laboratory of Plant Molecular Physiology, Graduate School of Agricultural and Life Sciences, University of Tokyo, TokyoJapan; ^3^Department of Advanced Bioscience, Faculty of Agriculture, Kinki University, NaraJapan; ^4^Institute of Plant Science and Resources, Okayama University, KurashikiJapan; ^5^PRESTO, Japan Science and Technology Agency, KawaguchiJapan

**Keywords:** transposon, small RNA, environmental stress, *Arabidopsis thaliana*, *ONSEN*

## Abstract

Transposable elements (TEs) are key elements that facilitate genome evolution of the host organism. A number of studies have assessed the functions of TEs, which change gene expression in the host genome. Activation of TEs is controlled by epigenetic modifications such as DNA methylation and histone modifications. Several recent studies have reported that TEs can also be activated by biotic or abiotic stress in some plants. We focused on a Ty1/copia retrotransposon, *ONSEN*, that is activated by heat stress (HS) in *Arabidopsis*. We found that transcriptional activation of *ONSEN* was regulated by a small interfering RNA (siRNA)-related pathway, and the activation could also be induced by oxidative stress. Mutants deficient in siRNA biogenesis that were exposed to HS at the initial stages of vegetative growth showed transgenerational transposition. The transposition was also detected in the progeny, which originated from tissue that had differentiated after exposure to the HS. The results indicated that in some undifferentiated cells, transpositional activity could be maintained quite long after exposure to the HS.

## INTRODUCTION

Plant genomes contain a large number of transposable elements (TEs) (Feschotte et al., 2002). In particular, retrotransposons that use RNA-mediated amplification constitute a large part of the plant genome (Kumar and Bennetzen, 1999). Retrotransposons are classified into two major subclasses on the basis of their sequence and structural similarity. The first class is long terminal repeat (LTR) retrotransposons, which contain LTRs on both sides. The second class is non-LTR retrotransposons, which include long interspersed nuclear elements and short interspersed nuclear elements (Schmidt, 1999). LTR retrotransposons are further divided into two families according to the order of the coding genes. Ty3/gypsy retrotransposons are enriched in intergenic regions that include centromeric heterochromatin in some plants (Jiang et al., 2003). The second family, Ty1/copia retrotransposons, are conserved in the euchromatic regions of some plant species (Kumar, 1996).

Although all plants contain various TEs, most of these are transcriptionally silenced through epigenetic regulation (Lippman et al., 2003). DNA methylation is one of the major regulators of transposon expression. TEs are activated in DNA hypomethylation mutants (Miura et al., 2001; Kato et al., 2003; Mirouze et al., 2009; Tsukahara et al., 2009), which represents a defense mechanism against ectopic transposon activation. One of the best-studied mechanisms for the regulation of TEs is RNA-directed DNA methylation (RdDM), which regulates TEs through small interfering RNA (siRNA)-mediated DNA methylation (Wierzbicki et al., 2008; Gao et al., 2010). Higher plants possess specific DNA-dependent RNA polymerases known as *RNA polymerase IV* (*PolIV*) and *RNA polymerase V* (*PolV*; Kanno et al., 2005; Onodera et al., 2005; Pontier et al., 2005). *PolIV* produces initial RNA transcripts for RNA silencing and *PolV* transcribes a messenger RNA for siRNA-induced DNA methylation on the target site.

In *Arabidopsis*, an ectopic transcript produced from TEs is transcribed to double-stranded RNA by *RNA-dependent RNA polymerase 2* (*RDR2*) and subsequently processed in 24–26 nt siRNAs by *DICER-LIKE 3* (*DCL3*; Zhang et al., 2007b; Mosher et al., 2008). The siRNAs bind to an RNA-induced silencing complex containing *ARGONAUTE 4* (*AGO4*) that interacts with *PolV* to recruit the DNA methyltransferase *DOMAINS REARRANGED METHYLTRANSFERASE 2* (*DRM2*), leading to *de novo* DNA methylation of the target TEs (Cao and Jacobsen, 2002; Matzke and Birchler, 2005).

It remains unknown when the TEs are activated or what triggers TE activation in nature. The answer may lie in accumulated evidence that TEs are activated by environmental stress. Some TEs were activated by abiotic stress in combination with DNA demethylation. For example, in corn, cold stress induced hypomethylation and activated the *ZmMI1* element that was similar at the LTR region of the putative retroelement (Steward et al., 2002).

In other cases, the activation of LTR retrotransposons is independent of DNA methylation and is under the control of *cis*-regulatory sequences in the LTRs. The best-characterized examples are the *Tnt1* and *Tto1* retrotransposons in tobacco. *Tnt1* and *Tto1* are activated by biotic stresses, such as tissue culturing, wounding, and pathogen infections, and by abiotic stresses, including salicylic acid and jasmonate (Peschke et al., 1987; Hirochika, 1993; Pouteau et al., 1994; Takeda et al., 1998). The stress-induced activation is controlled by *cis*-acting motifs in the U3 region of the LTR sequence that are similar to those of plant defense genes (Grandbastien et al., 1997; Mhiri et al., 1997; Vernhettes et al., 1997; Takeda et al., 1999).

Recently, a Ty1/copia retrotransposon named *ONSEN* was found to be activated by heat stress (HS) in *Arabidopsis* (Ito et al., 2011). The LTR of *ONSEN* contains a sequence that is recognized by the plant’s heat response transcription factor, *HsfA2* (Cavrak et al., 2014). *HsfA2* has a conserved N-terminal DNA-binding domain that binds a heat response element (HRE; Schramm et al., 2006). An electrophoretic mobility shift assay demonstrated that *HsfA2* bound to an HRE in the *ONSEN* LTR (Cavrak et al., 2014), indicating that *HsfA2* was directly involved in transcriptional activation. *ONSEN* is more strongly activated in a mutant that was deficient in a pathway for siRNA biogenesis than in the wild-type plant (WT), indicating that an siRNA-related pathway modulates transcriptional activation of *ONSEN* (Ito et al., 2011). Although heat-induced expression was enhanced by a mutant of DNA methyltransferase (Cavrak et al., 2014), *ONSEN* was not expressed in a hypomethylation mutant or a mutant lacking RdDM component without HS (Ito et al., 2011). This finding indicates that the initiation of the transcriptional activation is independent of DNA methylation.

In addition to transcriptional regulation, TEs may be controlled by transpositional processes in the host plant. A high frequency of new *ONSEN* insertions was observed in the progeny of stressed plants that were deficient in siRNA biogenesis (Ito et al., 2011). *ONSEN* was also expressed in the WT and in other epigenetic mutants subjected to HS; however, transgenerational transposition was not observed in the progeny (Ito et al., 2011). The results suggested that siRNA-mediated pathways regulated transgenerational transposition. Although the mechanism of *ONSEN* activation has been studied at the transcriptional level (Ito et al., 2011), the precise mechanism of transpositional regulation remains unknown. Here, we provide insight into siRNA-mediated regulation and the initiation of transgenerational transposition of *ONSEN*.

## MATERIALS AND METHODS

### PLANT MATERIAL AND GROWING CONDITIONS

The *Arabidopsis thaliana* plants used in the experiments included WT plants, *nrpd1* mutants (Herr et al., 2005), and transgenic plants that possessed a full-length LTR (genome position; 4212570–4213146) of *ONSEN* (*AT5G13205*) fused with a GFP gene. The plants were grown on Murashige and Skoog (MS) plates under continuous light at 21^∘^C. For the analysis of high light and oxidative stress, WT, *nrpd1*, and *hsfA2* T-DNA insertion mutant (Nishizawa et al., 2006) plants were grown on MS at 25^∘^C under continuous light (irradiance 100 μmol m^-2^ s^-1^). In order to analyze their reactions to short days and long days, plants ware grown on MS in pots for 7 days with continuous light at 21^∘^C and moved to a long-day chamber (16 h light and 8 h dark) or a short-day chamber (8 h light and 16 h dark). All WT and mutant plants were *A. thaliana* ecotype *Columbia*.

### STRESS TREATMENTS

For HS treatment, 7-day-old seedlings were subjected to a temperature shift from 21^∘^C to 37^∘^C for 24 h except some experiments. After undergoing heat treatment, plants were transplanted to soil and allowed to grow at 21^∘^C. For the high light and oxidative stress treatments, 7-day-old WT, *nrpd1,* and *hsfa2* plants were exposed to high light at 800 μmol m^-2^ s^-1^ at 25^∘^C or sprayed with 50 μM methylviologen.

### SOUTHERN BLOT ANALYSIS

*Arabidopsis* genomic DNA was isolated using a Nucleon PhytoPure DNA extraction kit (GE Healthcare Life Science). Blotting of genomic DNA was performed as described (Miura et al., 2004). Hybridization signals were detected using a radio-labeled *ONSEN*-specific probe (**Table 1**) that was generated with the Megaprime DNA Labeling System (GE Healthcare Life Science) in a high-SDS hybridization buffer (Church and Gilbert, 1984).

**Table 1:** Primer sequences.

Experiment	Primer	Sequence (5’-3’)
ONSEN probe	ONSEN-F	TAATGTTCCCTTCCAAGTCCC
	ONSEN-R	GCTTGTAATGACCCAAGAAGT
ONSEN RT-PCR	COPIA78-4129F_RT	CCACAAGAGGAACCAACGAA
	COPIA78-4300R_RT	TTCGATCATGGAAGACCGG
18S RT-PCR	18Sr-FW	CGTCCCTGCCCTTTGTACAC
	18Sr-RV	CGAACACTTCACCGGATCATT
GFP RT-PCR	GFP_qPCR_F	TGGCTTTGATGCCGTTCTTTTG
	GFP_qPCR_R	CGATTTCAAGGAGGACGGAAACAT
Cloning	AT5G13205-GFP-F1	CACCTGAGAAGCAGCAGAAACCAA
	AT5G13205-GFP-R1	AGGGAACATTGTTACTCGCCA

### REAL-TIME PCR

In order to analyze the TE expression resulting from heat treatment, total RNA was extracted from seedlings or leaves using TRI Reagent (Sigma T9424), according to the supplier’s recommendations. The five individual plants were pooled prior to RNA extraction. Around 3 to 5 μg of total RNA was treated with RQ1 RNase-free DNase (Promega) and reverse-transcribed using the ReverTraAce qPCR RT Kit (TOYOBO FSQ-101) with an oligo dT primer. In order to quantify the amount of *ONSEN* DNA, genomic DNA was extracted from seedlings or leaves using the DNeasy Plant Mini Kit (QIAGEN 69104), according to the supplier’s recommendations. Real-time PCR was performed using the Applied Biosystems 7300 Real Time PCR System with the THUNDERBIRD SYBR qPCR Mix (TOYOBO QPS-201). Three biological repetitions were performed and SD was determined (**Figure 2**). Similarly, three technical repetitions were performed and SD was determined (**Figures 4** and **10**). Following the high light and oxidative stress experiments, RNA was extracted using the Plant RNA Reagent (Life Technologies). A volume containing 2 μg of total RNA was subjected to RNase-free DNaseI (Thermo Scientific) and reverse-transcribed using the PrimeScript 1st strand cDNA Synthesis Kit (TaKaRa 6210A). Quantitative real-time PCR was performed with a LightCycler 96 System (Roche), using the FastStart Universal SYBR Green Master (ROX; Roche). Quantities of DNA were determined from a standard curve and were normalized to the amount of 18s rDNA. At least three biological repetitions were performed and SE were determined.

### LASER CAPTURE MICRODISSECTION AND RNA EXTRACTION

*Arabidopsis* seedlings were fixed in Farmer’s fixative (3:1 ethanol:acetic acid) overnight at 4^∘^C. Subsequently, fixation, dehydration, and paraffin infiltration were performed using a microwave processor (H2850 EBS). Paraffin-embedded sections were cut to a thickness of 12 μm and mounted on a PEN membrane glass slide (Applied Biosystems LCM0522). To remove the paraffin, slides were immersed in Histo-Clear II (National Diagnostics HS-202; 2 × 5 min) and then air-dried at room temperature. Laser capture microdissection was performed using the ArcturusXT LCM system (Applied Biosystems). Selected areas were captured by an infrared laser onto Arcturus CapSure Macro LCM Cap (LCM0211 Applied Biosystems) and subsequently cut with a UV laser. Total RNA was extracted using the PicoPure RNA Isolation Kit (Applied Biosystems KIT0204) and quantified using the Agilent RNA 6000 Pico Kit (Agilent 5067-1513).

## RESULTS

### HEAT ACTIVATION OF TRANSGENE

**FIGURE 1:**
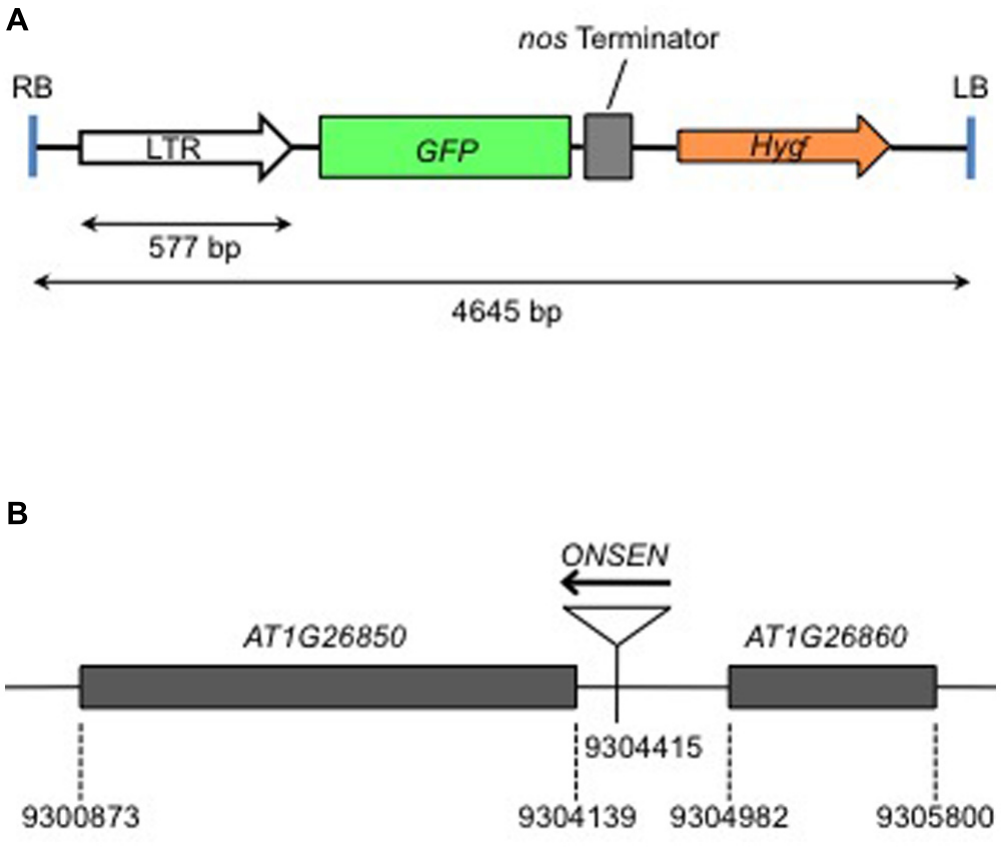
**Structure of the gene construct used to produce transgenic plants, and the insertion position of the transgene in the genome. (A)** The structure of the DNA sequence between the left border (LB) and the right border (RB) of the T-DNA. Intact long terminal repeat (LTR) of *ONSEN* (*AT5G13205*) was fused with the green fluorescent protein (*GFP*) gene. **(B)** The transgene was inserted in the intergenic region between *At1g26850* and *At1g26860*, in the same direction. Numerals indicate the nucleotide number according to the AGI map.

**FIGURE 2:**
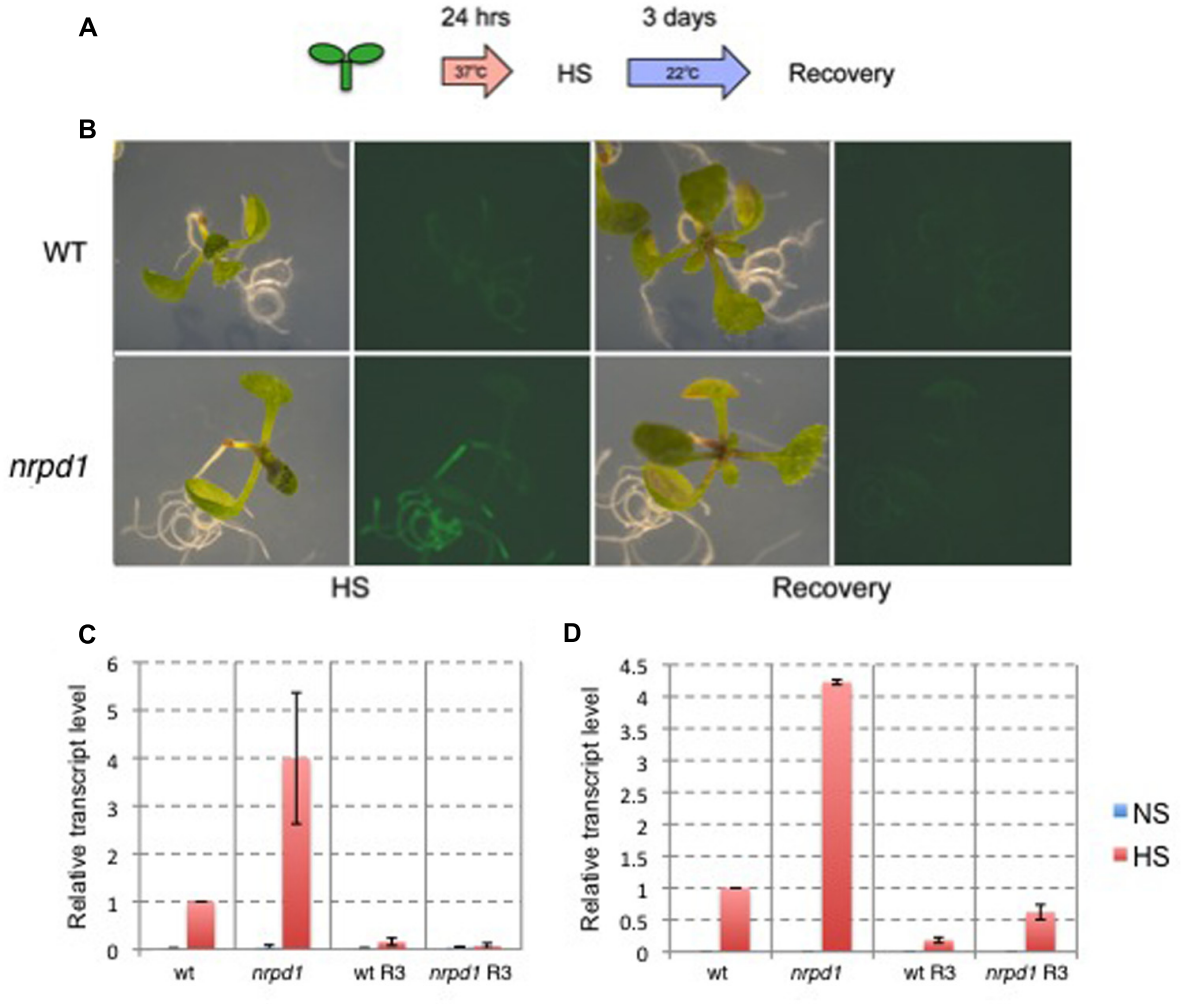
**Expression analysis of *GFP* driven by the *ONSEN* promoter. (A)** Scheme of the heat stress (HS) experiment. **(B)** Photographs of bright field and GFP fluorescence images. Wild-type plants (WT) and *nrpd1* plants (*nrpd1*) were observed immediately after HS and after the recovery phase (Recovery). **(C)** Relative transcription level of *GFP*. WT and *nrpd1* plants (*nrpd1*) were analyzed immediately after HS and after the recovery phase (R3). **(D)** Relative transcription level of endogenous *ONSEN*. The same samples as those used in c were used. NS, non-stress control samples. Error bars represent the mean ± SEM, *n* = 3; values are relative to heat-stressed wild-type (WT HS).

The heat-activation of *ONSEN* is controlled by the promoter in the LTRs that involves a *cis*-regulatory sequence for HREs (Cavrak et al., 2014). HREs are bound by the heat shock transcription factor (HSF) that controls heat-induced genes (Nover et al., 2001). We produced a transgenic *Arabidopsis* that possessed an intact LTR of *ONSEN* (*AT5G13205*) fused with a gene for green fluorescent protein (GFP) (**Figure 1A**). A transgenic plant with a single-copy insert was analyzed for expression of the reporter gene. The insertion was mapped on an intergenic region between *AT1G26850* and *AT1G26860* on chromosome 1 (**Figure 1B**). The GFP signal was analyzed immediately after a group of 10 1-week-old seedlings was subjected to heat treatment and again 3 days after the treatment (**Figure 2A**). The GFP signals were detected throughout the plants that were subjected to the heat treatment, but gradually decayed and were below the detection limit at 3 days after heat treatment (**Figure 2B**).

To understand the role of siRNA for transcriptional silencing of the transgene, the transgenic plant was crossed with *nrpd1*, a mutant that was deficient in siRNA biogenesis. The resulting transgenic line involved an LTR fused with *GFP* in an *nrpd1* background. The intensity of the GFP signal was stronger in the *nrpd1* transgenic line than in the WT line; however, the GFP signal fell below the detection level by 3 days after the heat treatment (**Figure 2B**). The intensity of the GFP signals was consistent with the transcription level of GFP (**Figure 2C**) and the endogenous *ONSEN* (**Figure 2D**). These results indicated that the transcriptional regulation of the transgene was controlled by an siRNA-related pathway that controls endogenous *ONSEN* copies.

### *ONSEN* ACTIVATION BY STRESS

**FIGURE 3:**
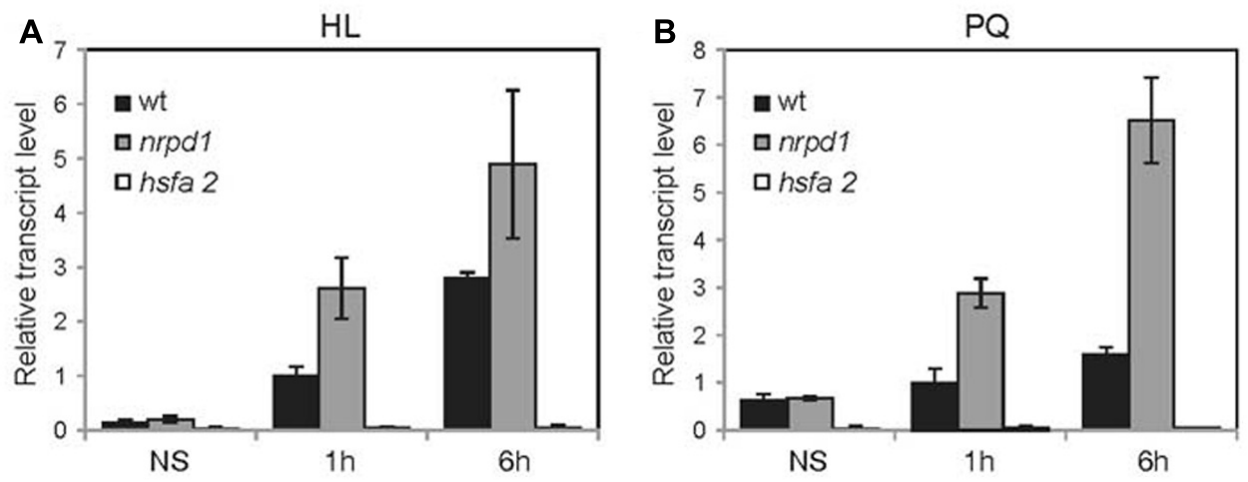
**Activation of *ONSEN* by high light stress and oxidative stress.** Plants before stress treatment, after continuous stress treatment for 1 h and after continuous stress treatment for 6 h were analyzed. **(A)** Relative transcription level of *ONSEN* immediately after high light stress (HL). **(B)** Relative transcription level of *ONSEN* immediately after Paraquat treatment (PQ). Error bars represent SDs (*n* = 3).

The LTR promoter of *ONSEN* contains a *cis*-regulatory sequence for HREs that binds to a heat-induced transcriptional factor, HsfA2 (Cavrak et al., 2014). HsfA2 was reported to play an important role in the response to environmental stresses, including high light stress (Nishizawa et al., 2006). To analyze the effect of high light stress on *ONSEN*, young seedlings were exposed to high light (800 μmol photons m^-2^s^-1^) for 6 h. The *ONSEN* mRNA level started to increase at 1 h and continued to gradually increase to the 6-h point, indicating that *ONSEN* was activated by high light stress (**Figure 3A**). The expression level of *ONSEN* was considerably higher in the *nrpd1* mutant than in the WT (**Figure 3A**), suggesting that the transcriptional activation was controlled by siRNA-mediated mechanisms.

To analyze the response of high light stress on *ONSEN* more precisely, we applied N,N^′^-dimethyl-4,4^′^-bipyridinium dichloride (Paraquat) to young seedlings. Paraquat produces hydrogen peroxide, which causes oxidative stress to the plant, similar to that caused by high light stress. Approximately 50 7-day-old seedlings were exposed to 50 μM Paraquat for 6 h. *ONSEN* was activated and at 6 h after the treatment its mRNA levels were increased (**Figure 3B**). In the *nrpd1* mutant, *ONSEN* was highly activated in response to oxidative stress (**Figure 3B**). Transcriptional activation was not detected in the *hsfa2* mutant that was subjected to neither high light stress nor to oxidative stress (**Figures 3A,B**). The results indicated that *ONSEN* was activated by oxidative stress via the HsfA2 transcriptional factor and that the activation was regulated by an siRNA-mediated pathway.

### THE QUANTITY OF ACTIVE *ONSEN* AFFECTED TRANSGENERATIONAL TRANSPOSITION

**FIGURE 4:**
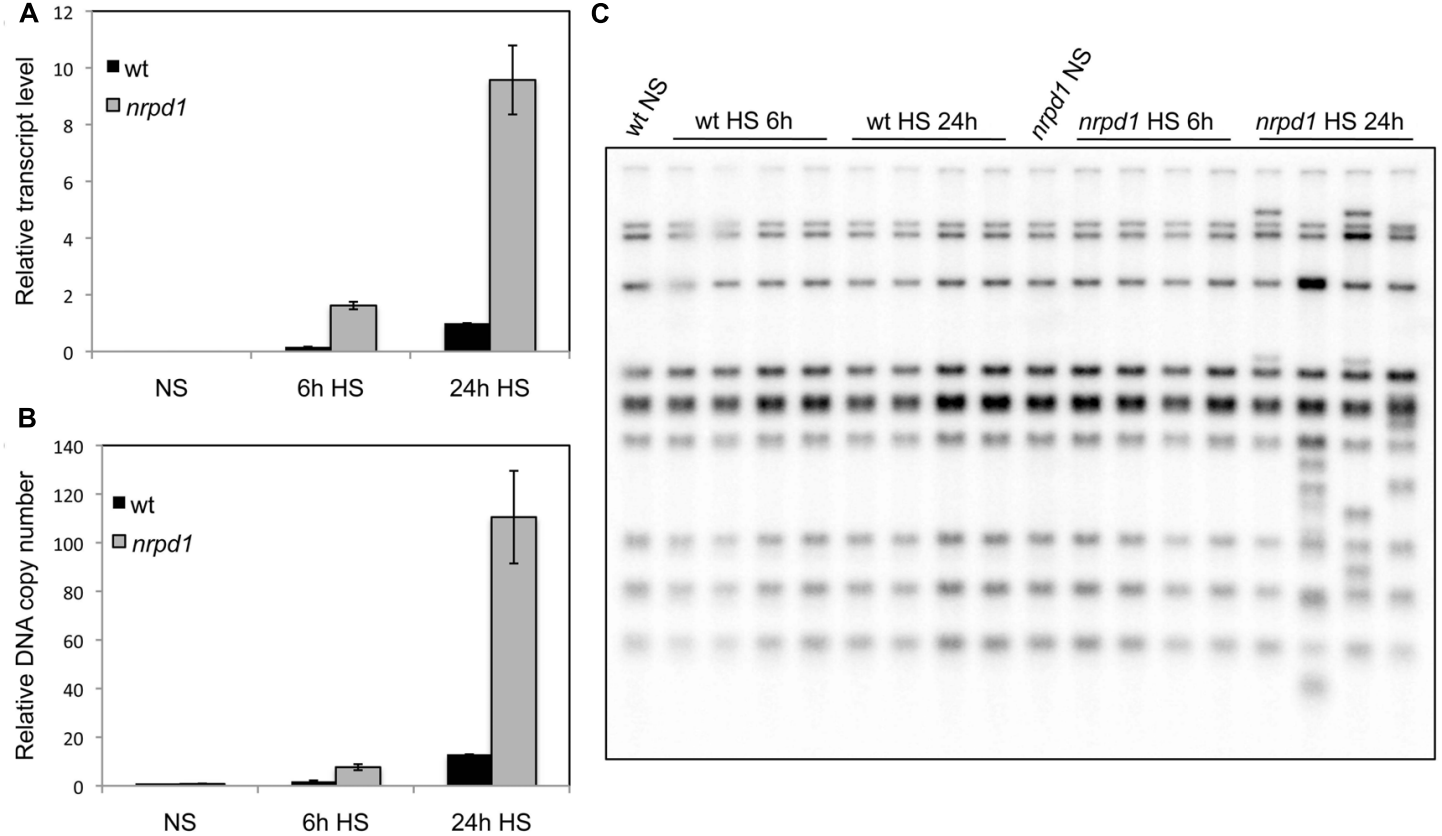
**The quantitative analyses of transcripts, extrachromosomal DNAs, and newly transposed copies in progeny.** WT and *nrpd1* plants were exposed to HS for 6 h and for 24 h at 7 days after germination. NS, non-stressed samples. **(A)** Relative transcription level of *ONSEN*. Error bar represents the mean ± SEM, *n* = 3, values relative to 24 h heat-stressed WT. **(B)** Relative number of copies of extrachromosomal DNA of *ONSEN*. Error bar represents the mean ± SEM, *n* = 3; values are relative to the NS WT seedlings. **(C)** Southern blot analysis of *ONSEN* in progeny plants.

To verify the effect of transcriptional activity and transposition frequency in *nrpd1*, 1-week-old seedlings were exposed to 37^∘^C for 6 h and 24 h, respectively. Quantitative analysis showed that after the 24-h HS, the transcription level had increased by approximately six times in both WT plants and *nrpd1* (**Figure 4A**). In addition, the number of synthesized extrachromosomal DNA copies was six times as high in the WT and 14 times as high in *nrpd1* plants (**Figure 4B**) as in these same seedling groups exposed to the 6-h treatment. New insertions of *ONSEN* were detected in the progeny of *nrpd1* that were subjected to HS for 24 h; however, they were not detected in the progeny of *nrpd1* that were subjected to HS for 6 h. A transposition was not detected in the progeny of WT plants subjected to HS (**Figure 4C**). These results suggested that a high amount of active *ONSEN* was important for transgenerational transposition to occur in the mutant that was impaired in the biogenesis of siRNAs.

### HEAT STRESS-INDUCED TRANSGENERATIONAL TRANSPOSITION IN SEEDLINGS

**FIGURE 5:**
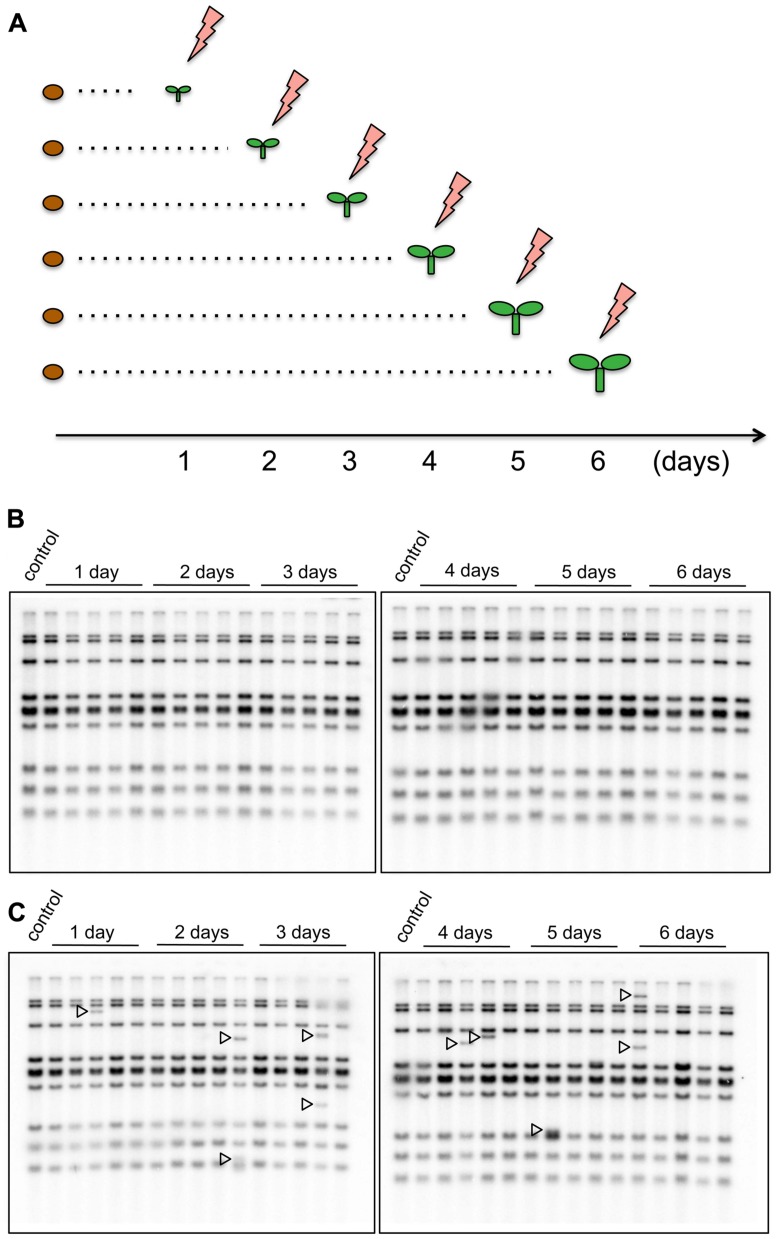
**Transgenerational transposition of *ONSEN* in young seedling. (A)** Scheme of the HS experiment. On each day after germination, seedlings were exposed to HS for 24 h. **(B,C)** Southern blot analysis of *ONSEN* using the progeny of WT **(B)** and *nrpd1* **(C)** plants that were exposed to HS, as indicated in **(A)**. Durations of HS are indicated over the lanes. NS plants were used as controls. Arrowheads indicate the transposed copies of *ONSEN*.

To investigate how the timing of the HS response in young seedlings affected transgenerational transposition, seedlings of the *nrpd1* and WT plants, at ages ranging from 1 to 6 days after germination, were exposed to 37^∘^C for 24 h (**Figure 5A**). Transgenerational transposition was analyzed in the progenies of heat-stressed plants. The new copies were not detected in the progeny of WT plants (**Figure 5B**), but they were detected in the progeny of stressed *nrpd1* plants (**Figure 5C**). Interestingly, transgenerational transposition was observed in seedlings whose parents were stressed at a very early developmental stage, as young as 1 day old (**Figure 5C**).

### TRANSGENERATIONAL TRANSPOSITION WAS INDEPENDENT OF FLOWERING TIME

**FIGURE 6:**
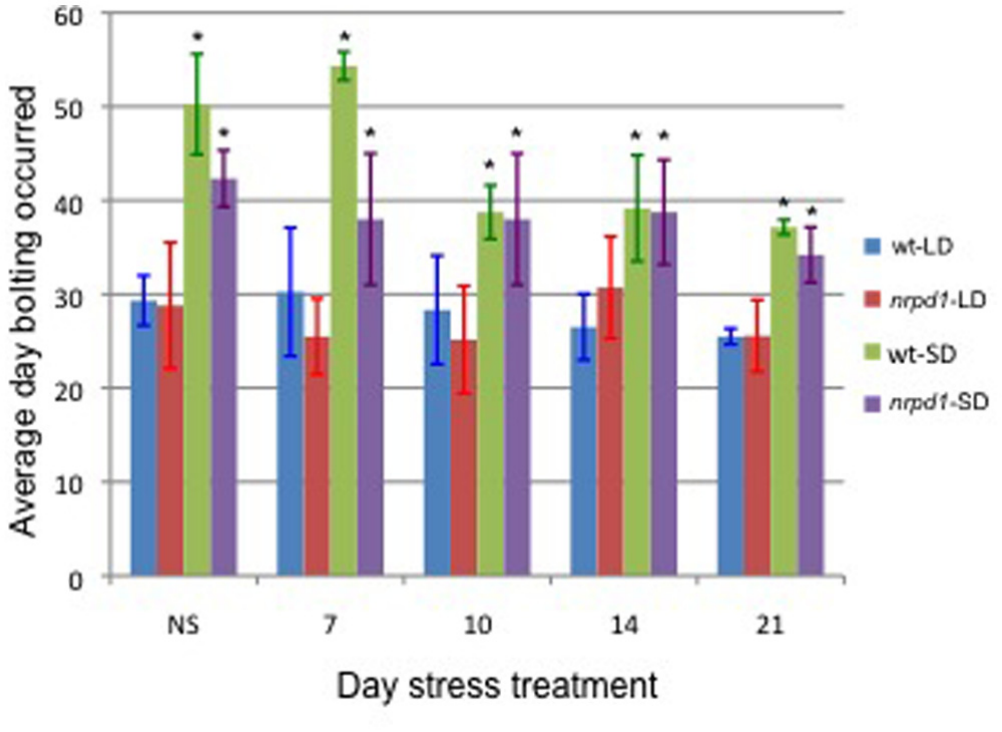
**The effect of daylight conditions on flowering time.** The average of the day on which bolting occurred was measured in 3 to 8 plants that grew under long-day (LD) or short-day (SD) conditions. Plants were exposed to HS for 24 h at 7, 10, 14, and 21 days after germination. The WT and *nrpd1* plants grew under LD and SD were compared, respectively, using a *t*-test. An asterisk indicates significantly different, *p* < 0.05. NS control plants.

Next, we analyzed whether transposition frequency was affected by flowering time. In *Arabidopsis*, flowering is controlled by photoperiod and is promoted by long day lengths. Under long-day conditions, bolting was observed 30 days after germination in both WT and *nrpd1* mutant plants. Under short-day conditions, bolting started approximately 50 days and 40 days after germination in WT and *nrpd1* plant, respectively (**Figure 6**).

**FIGURE 7:**
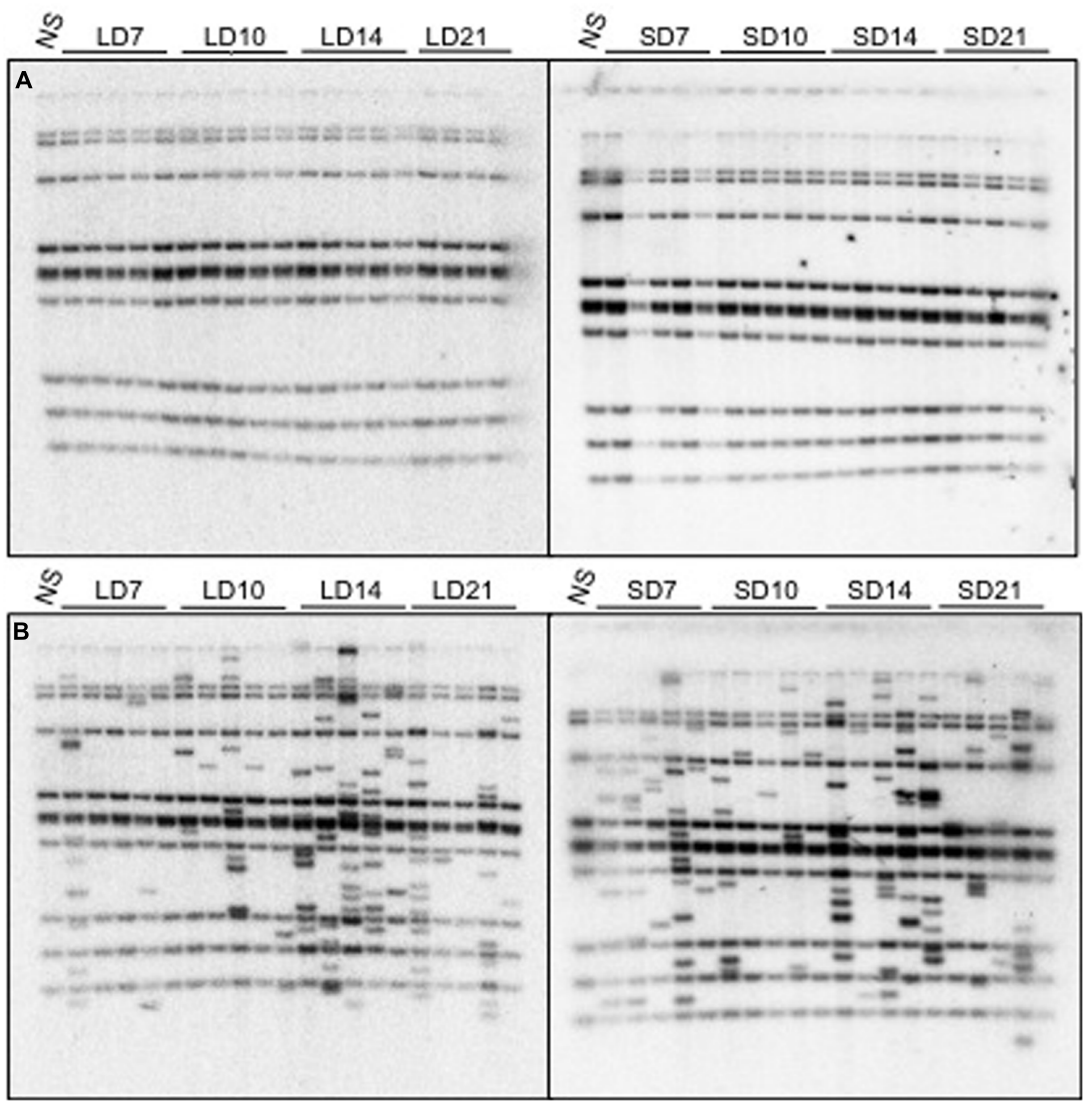
**Transgenerational transpositions in the WT **(A)** and *nrpd1***(B)** plants.** Data for plants grown under long-day (LD) and short-day (SD) conditions are shown in the left panels and right panels, respectively. 7, 10, 14, and 21 denote plants that were exposed to HS for 24 h at 7, 10, 14, and 21 days after germination, respectively. NS, non-stressed plants.

Seedlings that were growing under either long-day or short-day conditions were exposed to heat treatment 7, 10, 14, and 21 days after germination. The flowering time of the stressed *nrpd1* was slightly affected by the treatment (**Figure 6**). No new copy of *ONSEN* was detected in the WT progeny, which were growing under conditions that were neither long day nor short day (**Figure 7A**). Transgenerational transposition was observed in the progeny of *nrpd1* plants that were grown under either long-day or short-day conditions (**Figure 7B**). These results suggested that transposition frequency is not affected by flowering time and that the new insertions were transmitted into the reproductive tissue even when 3-week-old *nrpd1* plants were exposed to HS.

### STRESS MEMORY COULD BE REGULATED IN UNDIFFERENTIATED TISSUES DURING DEVELOPMENT

**FIGURE 8:**
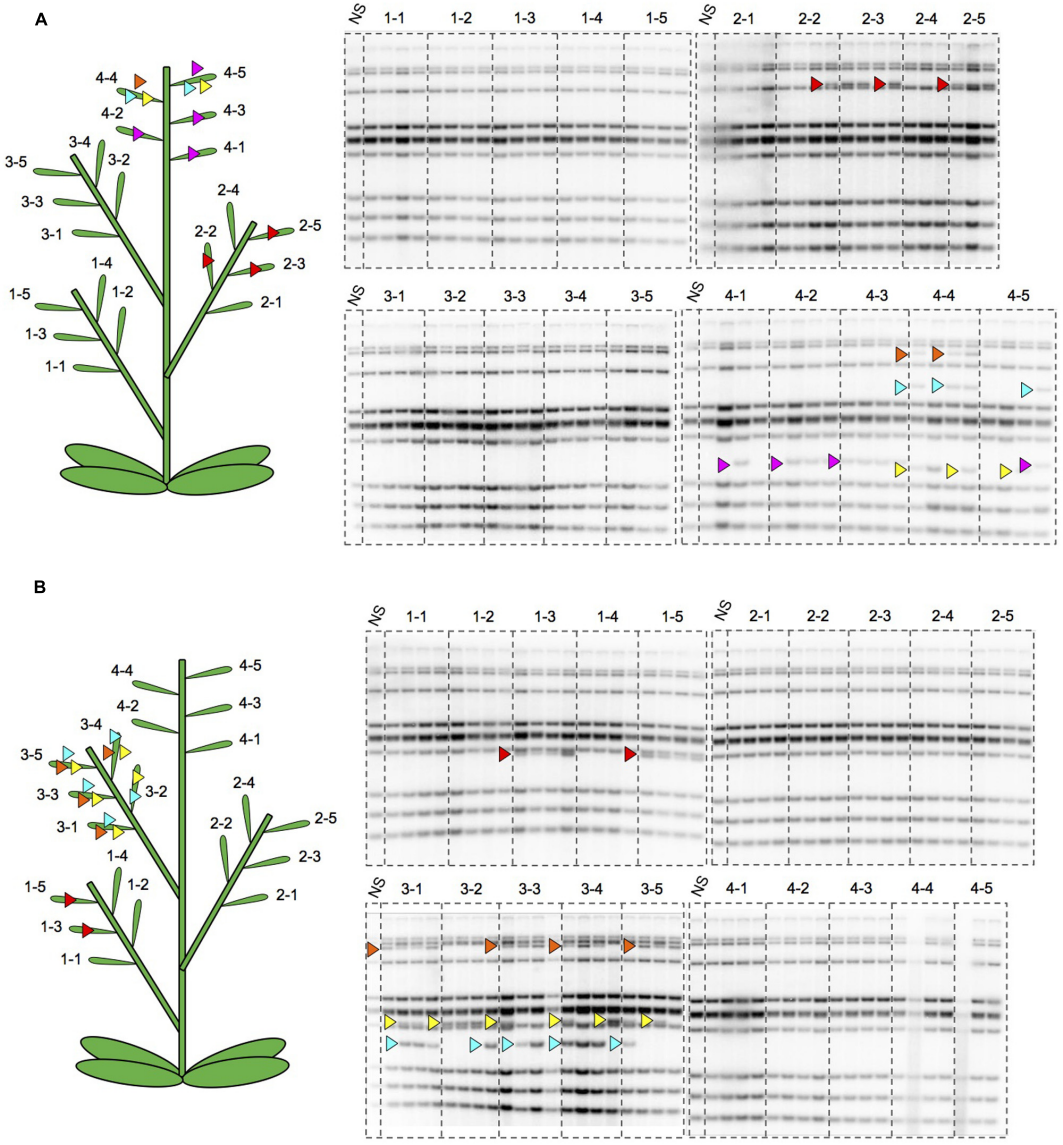
**Southern hybridization of *ONSEN* using the progeny of heat-stressed plants. (A)** DNAs were extracted from the progeny of one *nrpd1* plant. Numbers over lanes indicate the seed pods having seeds to grow progeny. Four progeny from the same seed pod were analyzed. The location of each seed pod on the parent plant is shown on the illustrated plant. Arrowheads indicate the new copies of *ONSEN* and the same color of arrowhead denotes that they transposed to the same loci. **(B)** DNAs were extracted from the progeny of another *nrpd1* plant. Symbols are the same as those used in **(A)**.

**FIGURE 9:**
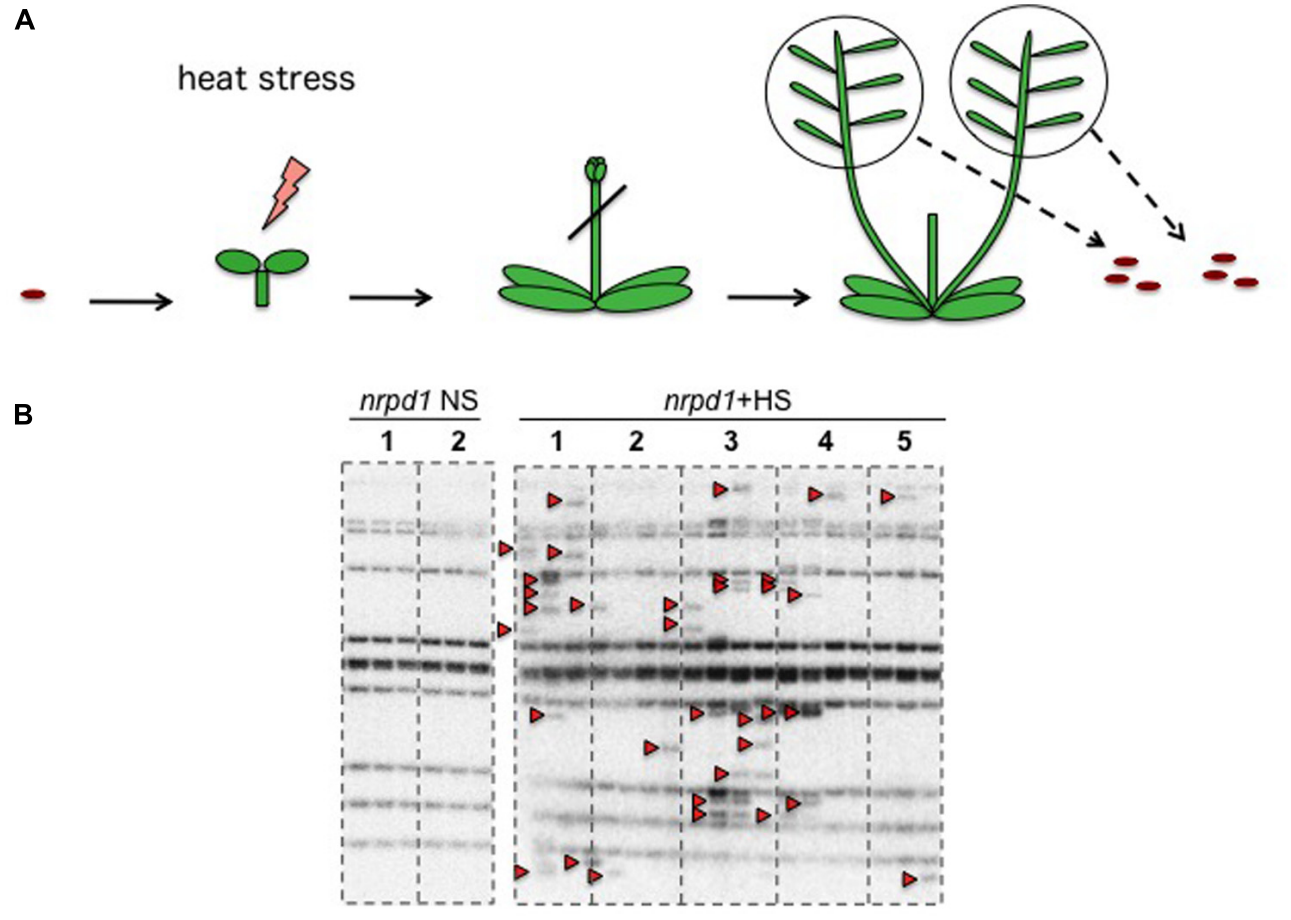
**Transgenerational transposition in late-differentiated branches. (A)** A scheme to obtain seeds for progeny. Parent *nrpd1* plants were exposed to HS for 24 h at 7 days after germination, then the primary flower stalk was cut immediately after bolting, and seeds were harvested from secondary branches. **(B)** Southern hybridization of *ONSEN*. DNAs of progeny from two NS parents and five HS parents were analyzed. Numbers over the lanes indicate that DNAs were extracted from the progeny of different parents. Arrowheads indicate the new copies of *ONSEN*.

To better understand the regulation of *ONSEN*, we observed new insertions of *ONSEN* in the siblings of a single flower on a single branch of a heat-stressed *nrpd1* plant. The pattern of new insertions was similar among the progeny within a single flower, although it differed among flowers on the same branch (**Figures 8A,B**). To understand the maintenance of heat activation of *ONSEN* during branch development, we analyzed transgenerational transposition in the secondarily produced branches. Surprisingly, new insertions were detected in an *nrpd1* progeny originating from the side shoots that was produced after cutting the initial shoot that was subjected to HS (**Figure 9**). This result indicated that the transposition activity could be maintained for a long period of time after HS.

### REGULATION OF *ONSEN* BY AN siRNA-RELATED MECHANISM IN UNDIFFERENTIATED TISSUES

**FIGURE 10:**
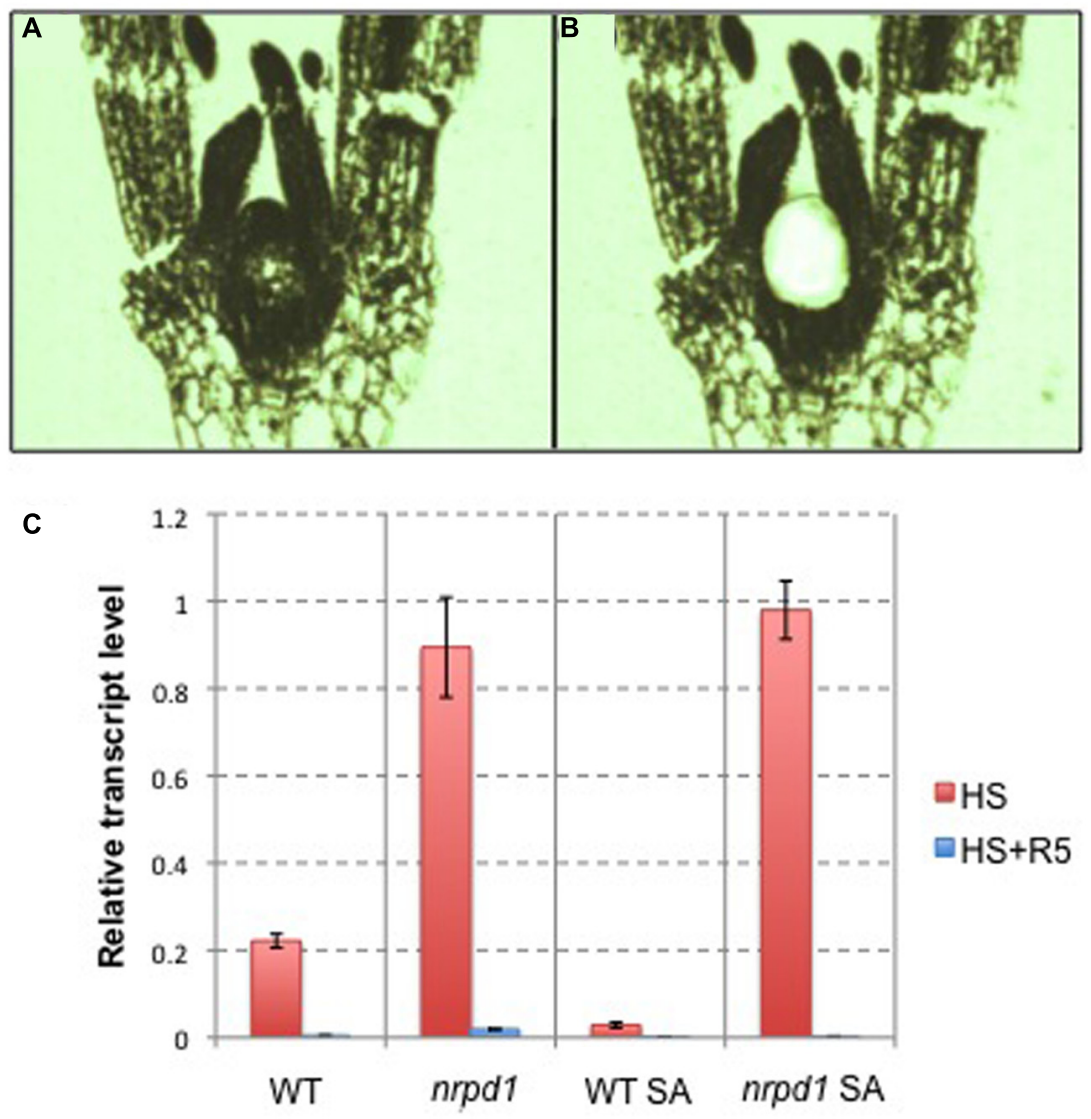
**Tissue-specific expression of *ONSEN*. (A)** A paraffin section of a young seedling. **(B)** A paraffin section of a young seedling after the shoot apex was isolated by laser capture microdissection. **(C)** Relative transcription level of *ONSEN* in young leaf tissue and the shoot apex immediately after being subjected to HS and 5 days after the heat-stressed (HS + R5). WT: hypocotyl tissue in WT plants. *nrpd1*: hypocotyl tissue in *nrpd1* mutant plants. WT SA: the shoot apex in WT plants. *nrpd1* SA: the shoot apex in *nrpd1* mutant plants. Error bars represent the mean ± SEM, *n* = 3; values are relative to heat-stressed WT.

To understand the mechanism of the transgenerational transposition of *ONSEN*, we analyzed the transcriptional activity of *ONSEN* in the shoot apex. The shoot apex, including the apical meristem, was fixed in paraffin (**Figure 10A**) and isolated by laser capture microdissection (**Figure 10B**). In *nrpd1* plants, *ONSEN* was highly activated in the shoot apex after heat treatment; however, the activity level returned to the baseline value 5 days after HS. In the WT plant, *ONSEN* was expressed at relatively low levels even after heat treatment (**Figure 10C**). These results indicated that an siRNA-related pathway regulated transcriptional activation of *ONSEN* in the shoot apex.

## DISCUSSION

Transposons are abundant in plant genomes and show great diversity among species. Large numbers of transposon copies may occur in a host genome as a result of activation of TEs by an external stimulus or environmental stress (Wessler, 1996; Grandbastien, 1998; Capy et al., 2000). We analyzed a Ty1/copia retrotransposon, *ONSEN*, as a model for stress-activated transposon. *ONSEN* is a relatively young element with conserved LTR sequences and functional coding genes in *Arabidopsis* (Ito et al., 2011). The LTR sequence contains conserved HREs that become functional stress-responding promoters of *ONSEN* (Cavrak et al., 2014). It is not known how *ONSEN* acquired the stress-responding promoter in the LTR; however, after a TE has gained a functional promoter that responds to environmental stress, the number of copies of activated TE may be amplified in the host genome when environmental changes occur in nature.

We analyzed the heat-induced promoter of *ONSEN* using a reporter gene assay. Gene expression was enhanced in a mutant that was deficient in siRNA biogenesis. Reduction of DNA methylation in the LTR has been shown to favor the HS response (Cavrak et al., 2014). However, we showed that the activity level of the transgene gradually declined and was no longer detected after 3 days, indicating that the activation of *ONSEN* was initially regulated by transcriptional factors and that the resilencing of *ONSEN* was controlled independently of RdDM. In *Arabidopsis*, heat-activation of several repetitive elements can occur without epigenetic changes such as DNA methylation and histone modifications but is accompanied by heterochromatin decondensation (Pecinka et al., 2010). The transcriptional silencing of the activated elements delayed in mutants of CAF-1 which loads nucleosomes onto replicated DNA suggested that CAF-1 was important for efficient restoration of silencing after HS (Pecinka et al., 2010). Recently, it is reported that a chromatin-remodeling factor, Decrease in DNA Methylation 1 (DDM1) and Morpheus Molecule 1 (MOM1) act redundantly to restore silencing after HS in *Arabidopsis* (Iwasaki and Paszkowski, 2014). The heat-induced transcriptional activation of some genes in *ddm1mom1* double mutants persisted longer than in WT and was transgenerationally inherited. These findings indicated that heat-induced chromatin changes might play a role for the rapid resetting of *ONSEN* expression in *nrpd1* with impaired siRNA repression.

Transposable elements could be activated in a harsh environment and could affect the host genome. For example, in *Arabidopsis*, activated *Athila* retrotransposons produce a small RNA that regulates a stress-response gene, *OLIGOURIDYLATE BINDING PROTEIN 1* in trans (McCue et al., 2012). We determined that *ONSEN* was activated not only by HS but also by oxidative stress. Activity levels were enhanced in the mutant that was deficient in siRNA biogenesis, indicating that the activation was controlled by RdDM. The activation of *ONSEN* was not detected in the mutant lacking heat-inducible transcription factor, suggesting that activation of *ONSEN* induced by oxidative stress is regulated by the same pathway as heat-induced activation.

The mobility of *ONSEN* was regulated tightly by the siRNA-mediated pathway. In a mutant of siRNA biogenesis, a transgenerational transposition was observed, indicating that *ONSEN* transposition was transmitted to a reproductive cell. In this report, we analyzed whether transcriptional activation timing was an important factor for transposition frequency during plant development. Transposition occurred when the plant was heat-stressed during a period of the developmental phase. The same transposition pattern on the progeny within a single flower indicated that some transposition occurred prior to flower differentiation. Further, the same transposition pattern among the progenies from different branches indicated that some transposition occurred before branch development. Conversely, the different transposition pattern in the progeny of adjacent flowers indicated that transposition occurred just before flower bud initiation. The observation that transposition occurred in the progenies produced from flowers that were differentiated after HS indicated that transposition activation might be maintained over a long period of time, although the transcriptional activation was transient.

One question that remains to be resolved concerns the regulatory mechanism of the transposition that occurred during the period of flower differentiation. One possibility is that transposition requires HS during the period of inflorescence meristem formation. We found that transposition occurred when HS was applied to 1 day-old seedling in which the inflorescence meristem has not been formed, suggesting that the presence of inflorescence meristem cells is not necessary for HS-induced transposition. Another possibility is that the activity levels of *ONSEN* may be maintained in the shoot apical meristem and transposition may have occurred at the point when the cell’s fate changes during the reproductive growth of a stressed mutant. A further possibility is that *ONSEN* RNA was maintained in the shoot apical meristem during plant development. We found that the transcriptional activity levels of *ONSEN* returned to baseline levels within 5 days in the shoot apex of both WT and *nrpd1* mutant plants. This result indicated that neither the activity level of *ONSEN* nor *ONSEN* RNA was maintained in the shoot apex during plant development. We could not exclude the possibility that the transcriptional activity of *ONSEN* was sustainable only in specific tissues, such as the shoot apical meristem of *nrpd1* plants.

The transposition of *ONSEN* during flower differentiation might require an active mark in addition to the transient activation by HS. The transposition of *ONSEN* was independent of DNA methylation and *ONSEN* was not transposed in *ddm1* hypomethylation mutation subjected to HS (Ito et al., 2011). The HS might induce active chromatin in *ONSEN*; however, subsequently, siRNA could induce *ONSEN* as a silenced chromatin. A transgenerational transposition of heat-activated *ONSEN* was not observed in a mutant deficient in histone H3K9 methyltransferases (*SUVH4/KRYPTONITE*) (Ito et al., 2011), indicating that the regulation of transposition was independent of histone H3K9 dimethylation. Histone H3K27 trimethylation (H3K27me3) is associated with gene repression and the target genes are tissue-specifically activated during differentiation processes or induced by abiotic or biotic stresses in *Arabidopsis* (Zhang et al., 2007a). H3K27me3 dynamically regulated the target gene expression during plant differentiation and the targets were enriched in TEs in meristem indicating that stem cells must be protected from TE activation to form germline (Lafos et al., 2011). *ONSEN* was a target of H3K27me3, however, the methylation level was not significantly different in meristem and leaves in WT (Lafos et al., 2011). It is interesting to know whether HS in *nrpd1* mutant could affect the modification of H3K27me3 on *ONSEN* in a tissue-specific manner.

In *nrpd1* mutant plants, HS could change chromatin structures, thereby releasing *ONSEN* transposition, and the active chromatin state could be maintained until the period of flower differentiation. A large-scale reorganization of chromatin was observed during floral transition in *Arabidopsis* (Tessadori et al., 2007). The pericentric heterochromatin reduced prior to bolting and recovered after elongation of the floral stem. Also decondensation of chromatin in gene-rich regions coincided with the floral transition. We still do not know the meristem-specific regulation of transposition during floral transition; however, transposon silencing in plant germ cells has been reported in *Arabidopsis* (Slotkin et al., 2009; Olmedo-Monfil et al., 2010; Ibarra et al., 2012). It is worth noting that chromatin decondensation was not observed in nuclei from meristematic tissue after HS (Pecinka et al., 2010). Recently, it has been reported that RdDM is function specific in the shoot apical meristem and reinforces the silencing of TEs during early vegetative growth (Baubec et al., 2014). These findings demonstrated the importance of TE regulation during vegetative growth prior to the formation of the next generation.

It is also worth noting that the progeny of heat-stressed *Arabidopsis* had fewer but large leaves and tended to bolt earlier to increased plant biomass (Migicovsky et al., 2014). In the stressed progeny, the expression of *HsfA2* and *ONSEN* was elevated with decreasing of global DNA methylation. The transgenerational phenotypic and epigenetic changes were partially deficient in the Dicer-like mutant, however, *ONSEN* expression increased in the progeny of heat-stressed plants regardless of mutant type (Migicovsky et al., 2014). It will be worthwhile to investigate the mechanism by which the stress memory is maintained during plant development.

In this study, siRNA-mediated regulation functioned to control transposons that were ectopically activated by environmental stress. Plant has evolved the regulation mechanisms that were independent of suppression by constitutive heterochromatin. The regulation was important during the process of plant development and may have a function in undifferentiated cells.

## Conflict of Interest Statement

The authors declare that the research was conducted in the absence of any commercial or financial relationships that could be construed as a potential conflict of interest.
